# Physiological and cognitive military related performances after 10-kilometer march

**DOI:** 10.1186/2054-314X-1-6

**Published:** 2015-02-24

**Authors:** Ran Yanovich, Amir Hadid, Tomer Erlich, Daniel S Moran, Yuval Heled

**Affiliations:** 8 Warrior Health Research Institute, IDF Medical Corp, Tel Hashomer, Ramat-Gan, Israel; 9 Heller Institute of Medical Research, Sheba Medical Center, Tel Hashomer, Ramat-Gan, Israel; 10 Ariel University Center of Samaria, Ariel, Israel

**Keywords:** Pre-mission activities, Time to exhaustion, Four choice reaction time, Visual vigilance task

## Abstract

**Background:**

Prior operational activities such as marching in diverse environments, with heavy backloads may cause early fatigue and reduce the unit’s readiness. The purpose of this preliminary study was to evaluate the effect of 10-kilometer (km) march on selected, military oriented, physiological and cognitive performances.

Eight healthy young males (age 25 ± 3 years) performed a series of cognitive and physiological tests, first without any prior physiological strain and then after a 10 km march in comfort laboratory conditions (24°C, 50%RH) consisting a 5 km/h speed and 2-6% incline with backload weighing 30% of their body weight.

**Results:**

We found that the subjects’ time to exhaustion (TTE) after the march decreased by 27% with no changes in anaerobic performance. Cognitive performance showed a significant (20%) reduction in accuracy and a tendency to reduce reaction time after the march.

**Conclusions:**

We conclude that a moderate-intensity march under relatively comfort environmental conditions may differently decrease selected military related physical and cognitive abilities. This phenomenon is probably associated with the type and intensity of the pre-mission physical activity and the magnitude of the associated mental fatigue. We suggest that quantifying these effects, as was presented in this preliminary study, by adopting this practical scientific approach would assist in preserving the soldiers’ performance and health during training and military operations.

## Background

Combative military missions require maximal physiological, cognitive and mental performances [1]. In many cases, soldiers are required to act after marching in diverse terrains and carrying heavy back loads. These combined factors add significant physiological burdens on the soldiers and may cause physical and mental exhaustion before reaching the primary mission. It is therefore a crucial and practical matter that requires careful scientific attention. Measurements of physical performances after various types of physical loads have been previously described [2]. However, more data on military related scenarios and performances are required. Mapping the influence of various pre-mission physical activities on the soldier’s performance may assist in making evidence-based decisions regarding the unit’s physical condition, where the main goal is to preserve the soldiers’ function and health.

The most extreme physical and mental load that the soldier may experience occurs during complex mobile multitask military outline in harsh topographical open-field mission [3]. During these conditions, the dominant physical fitness components are speed, agility, explosive power, flexibility, and endurance. From the cognitive perspective, the soldier is required to be vigilant, fast in decision making and reaction time and to have maximal concentration. These functions may be negatively affected during pre-mission activities and thereby later influence the soldier’s performance during the operational mission.

The purpose of this preliminary laboratory study was to measure the effects of a typical pre-mission physical activity scenario on diverse physiological and cognitive military related functions.

## Methods

### Subjects

Eight healthy, fit, young male volunteers (age 25 ± 3 yrs; height 177 ± 5 cm; weight 72 ± 12kg, VO_2max_ 56 ± 4 ml · min^-1^ · kg^-1^) who served in combat military units and currently serve as reserve soldiers in similar units participated in this study. Each subject signed an informed consent prior to participation and went through a medical examination. The study protocol was approved in advance by both the Sheba Medical Center IRB-Helsinki Committee (International Review Board for human and animal trials) and the IDF Human Use committee.

### Study protocol

The study was performed in laboratory conditions and consisted of three phases, where each phase took place on a separate day. In general, as for dependent variables, physical and cognitive performances were selected for the study, and the independent variable of the study was controlled military marching in controlled comfort climatic conditions. All the tests were performed during similar time of the day (mornings). Three to five days of rest were given between the phases. All the protocols were performed in relatively comfort environmental conditions (24°C, 50%RH). The subjects were instructed to avoid any extreme physical activities throughout the entire study period and any exercise during the 48 hours before participation in any of the exercise tests. They were given similar general instructions regarding nutrition, and specific instructions regarding their meals prior to arrival in the lab. The subjects consumed 500 ml of water one hour before starting each of the protocols. Moreover, they were instructed not to drink coffee or to smoke in the mornings prior to arriving in the lab, not to drink alcohol for at list 24 hours before participating in each one of the study phases, and to sleep at list 7 night hours before arriving to the lab. The importance of fulfilling these instructions was continuously emphasized throughout the entire study and we verified it before starting each one of the study phases. The three phases (experimental days) included the following protocols:

Phase 1 (day 1) - Maximal oxygen uptake (VO_2max_) and onset of blood lactate accumulation (OBLA) were measured. The tests were performed using online computer assisted open-circuit spirometry (Sensor Medics, Yorba Linda, CA, USA). The VO_2max_ was determined during standard modified incremental exercise on a motorized treadmill [4]. The protocol started with 3 min of warm-up walking at 5 km/h. The treadmill speed was then increased to 7 km/h, and then by 1 km/h every 3 min. until volitional fatigue. The treadmill speed was subsequently lowered to 5 km/h for an active recovery. Valid maximal oxygen consumption was accepted when at least three of the following criteria were met: *1*) a plateau in oxygen consumption with increasing work rate; *2*) a respiratory exchange ratio at maximal exercise of >1.10; *3*) achievement of age-predicted maximal heart rate (HR; 220 – age); and *4*) subjective exhaustion- the subject’s request for ending the test. Blood sample for lactate was taken every 3 minutes before increasing the treadmill’s speed using the capillary method and analyzed using lactate analyzer (Roche Diagnostics, Germany). OBLA was defined when blood lactate concentration was ≥4 mmol/l [5].

Phase 2 (day 2) - A series of cognitive and physical fitness tests were performed in order to determine the subjects’ baseline functions. The cognitive tests included two tests that were performed using unique computerized laptop software: the Visual Vigilance Task- VVT [6] and the Four Choice Reaction Time- FCRT [7]. To note, the subjects went through a training period starting three days before the study until reaching a plateau in their cognitive tests’ results in order to avoid a learning process during the study. The VVT’s aim was to evaluate the subject’s visual awareness, which is an important expression of vigilance. During the test the subject viewed a computer monitor with a dark background and was required to respond when a small rectangle appeared. Responses were made by pressing the space bar of the keyboard and the reaction time was then measured. The stimuli remained on the screen for 2 sec. A space bar pressed during this time was considered a hit. Failure to do so was scored as a miss. Pressing the space bar at any other time counted as a false alarm response or an incorrect identification. The total test duration was set to 5 minutes and the delay between stimulus occurrences was random.

The FCRT was also administered on a computer monitor and evaluated a more complex reaction time mission. When a black circle emerged in a field on the screen, the subject was supposed to press the corresponding key on the keyboard as quickly as possible. The next circle emerged after 500 msec and a total of 52 black circles were exposed. The time to complete the test and the number of errors were recorded. The total test duration was also set to 5 min.

The next step in Phase 2 consisted of a battery of five physical fitness tests. The tests were performed while wearing military uniforms in the following order:

1. **Velocity test**- included two sessions of 30 meters maximal speed running with a 1 min. rest between the sessions. The average time of the two sessions was calculated.
2. **Agility test**- included two sessions of 4×10 meters maximal speed running with a 1 min. rest between the sessions. The average time of the two sessions was calculated.
3. **Upper body endurance test**- included maximal number of push-ups.
4. **Lower body explosive power test**- included three standing long jumps. The average jumping distance was calculated.
5. **Time to exhaustion test (TTE)** - This test was performed after a 1 hr rest from the four fitness tests described above. The TTE test consisted of walking on a treadmill wearing a military backpack weighing 30% of the subject’s body weight, as accepted during operational missions [8] in comfort environmental conditions (24°C, 50%RH). After a 2 min. warm-up (5 km/h, 2% slope), the pace was increased to 5.5 km/h and the slope to 7.5% for 17 min., and then to 6 km/h and 8%, respectively, until reaching a subjective exhaustion. The subjects’ heart rate (HR) was continuously monitored using Polar watch with chest belt (Polar Electro, USA). Blood lactate was measured prior to the beginning of the test and immediately after stopping the test due to exhaustion.

Phase 3 (day 3) - The same battery of physical and cognitive tests from phase 2 was performed, this time after a 10 km march. The march was performed inside an environmental chamber in comfort environmental conditions (24°C, 50%RH) on a treadmill at a speed of 5 km/h and 2% slope while carrying backpack weighing 30% of body weight. In order to simulate diverse terrain, the treadmill slope was increased to 6% for 5 min. every 20 min. during the march. After the first hour (from a total walking time of 2 hours) the subjects sat for 10 minutes. The two cognitive tests (10 min. total) were performed immediately after the march, followed by the battery of previously described physical fitness tests (15 min.) and then by the TTE test. We then compared the subjects’ performances at baseline to those that were measured after 10 km march.

### Statistical analysis

In order to assess the effect of the prior march on both cognitive and physical performances, we used the student’s one-sample paired *t*-test, where each subject served as his own control. The alpha level was set at the 0.05 level and the pre-specified level of statistical power for calculating the sample size was set to 80%. The results were presented as average ± standard errors (SE). We used the Excel 2010 data analysis package.

## Results

**Figure 1:**
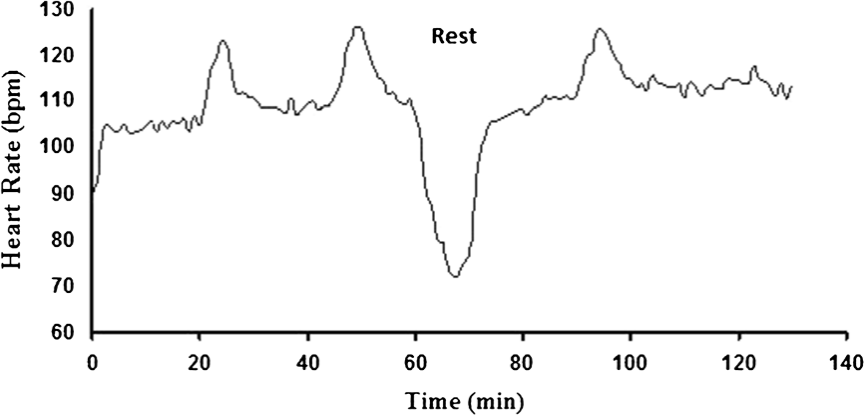
**Average heart rate (HR) (N = 8) during the march (Phase 3) in comfort environmental conditions (24°C, 50%RH).**

**Figure 2:**
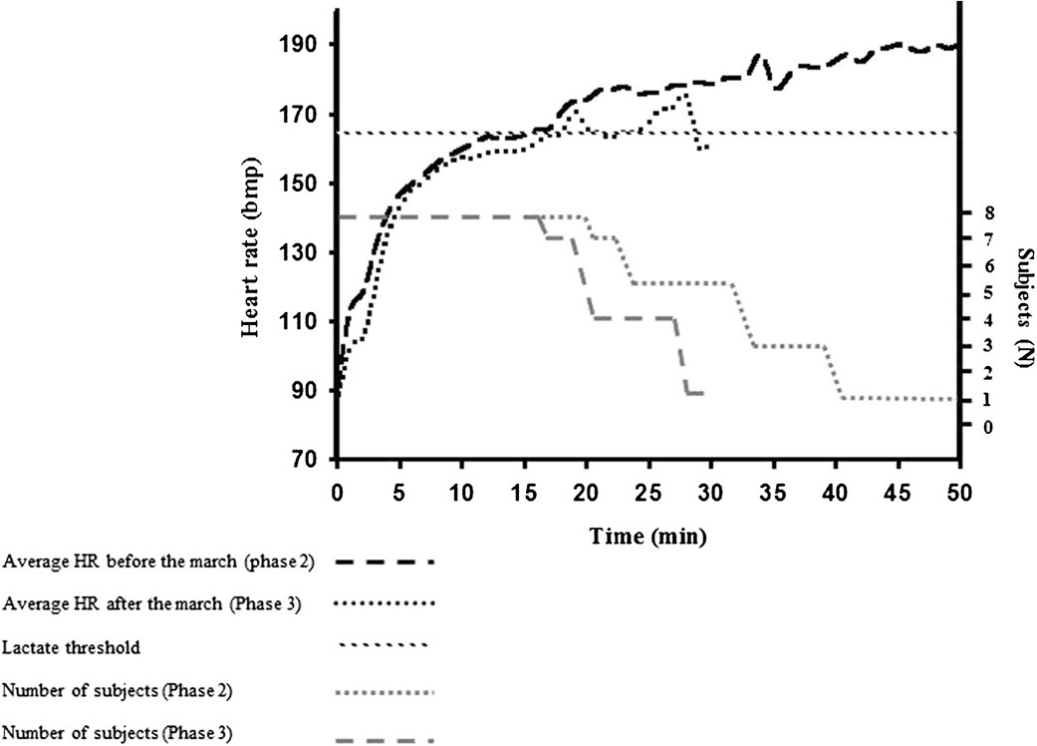
**Average heart rate (HR) dynamics (N = 8) and dropout during the Time to exhaustion (TTE) test at baseline (Phase 2) and after the march (Phase 3).**

The average HR dynamics during the march are presented in Figure 1. During walking at a pace of 5 km/h and 2% slope, the average HR was 110 ± 5 bpm, and when the slope was increased to 6%, the average HR increased by 15.5% to 127 ± 6 bpm. The average time to exhaustion during the TTE test at baseline was significantly longer (by 27%) compared to that measured following the march (32 ± 10 min. and 23 ± 5 min., respectively) (p < 0.05). In addition, subjective exhaustion started after 22 min. compared to after 17 min., respectively (Figure 2). We performed the Log-rank test to compare the survival curves of time to exhaustion between baseline (before the march) and after the march. The results were statistically significant (p = 0.016) with medians being 37 and 20 min. for the baseline and post-march groups, respectively.

During the TTE test that followed the march the average HR was higher by 9 ± 3 bpm compared to that measured during the baseline test (156 ± 4 bpm compared to 147 ± 5 bpm, respectively) (p < 0.05). These HR results, however, only present the first 17 min. of the TTE tests, since starting from the 17^th^ min, subjects began to drop out of the TTE test performed following the march due to exhaustion, and further valid statistical analyses could not be performed. The average maximum HRs at the exhaustion point during the TTE tests were 185 ± 6 bpm and 181 ± 8 bpm in the baseline and in the post-march tests, respectively.

Blood lactate level at the end of the 10 km march was 3.00 ± 0.51 mmol · l^-1^ and increased in average of 6.64 ± 2.54 mmol · l^-1^ at the exhaustion point of the TTE test that followed the march (p < 0.05). During the baseline TTE test exhaustion point blood lactate remained the same 6.90 ± 3.6 mmol · l^-1^. OBLA during the baseline TTE test reached the average point after 16 ± 2 min, compared to after 7 ± 1 min. when the test was performed following the march (p < 0.05).

The battery of anaerobic physical fitness tests results is presented in Table 1. As can be seen, no significant differences were found between the tests when performed at baseline and after the march.

**Table 1:** **The physiological tests results (maen ± SD) in Phase 2 (baseline) and Phase 3 (after the march)**

Test	Phase 2	Phase 3	p-value
**Velocity test (sec)**	4.93 ± 0.21	4.99 ± 0.19	NS
**Agility test (sec)**	10.41 ± 0.28	10.92 ± 0.71	NS
**Push-ups (rep)**	49 ± 22	50 ± 26	NS
**Standing long jump (cm)**	227.66 ± 13.88	224.11 ± 21.18	NS

The average VVT reaction time at baseline and following the march was not different (1.024 ± 0.12 sec vs. 1.068 ± 0.15 sec, respectively, p = NS). Nevertheless, the number of correct responses after the march vastly decreased by 20% compared to baseline (12 ± 5 vs. 15 ± 4, respectively, p < 0.05).

The average FCRT reaction time after the march was not different compared to baseline (529 ± 79 msec vs. 535 ± 78 msec, respectively, p = NS), but the average score of the correct answers tended to decrease, although not significantly (463 ± 27 correct answers vs. 480 ± 17 correct answers, respectively; p = NS).

## Discussion

The main purpose of this preliminary study was to measure changes in several key physiological and cognitive functions after a 10 km march performed in laboratory conditions. We suggest that evaluating the soldier’s performance changes after various pre-mission activities or during training will assist in predicting his physical and operational capabilities and will contribute to preserving his function and health.

The results showed that anaerobic abilities (velocity, agility, lower limbs explosive power, and upper limbs endurance) did not significantly change after a 10 km march with backpack weighing 30% of body weight performed in comfort environmental conditions. We suggest that due to relatively low intensity of the prior march as was expressed by both relatively low average HRs (110 ± 5 bpm at the 2% slope and 127 ± 6 bpm at the 6% slope) and low blood lactate levels at the end of the march (3 ± 0.51mmol · l^-1^), anaerobic explosive performances that are mostly dependent on the alactic energy system (ATP-PCr) were not reduced. Moreover, as for the push-ups test, the fact that the prior march did not include any upper body exercise, could also contribute to these results. However, more intense, long activities may limit these functions due to general and local fatigue and reduction in available energy.

On the other hand, the effect on endurance aerobic performance was noteworthy (p < 0.05) as was expressed by both a 56.3% reduction in time to OBLA during the TTE test that followed the march, and by a higher average HR throughout the test (by 9 ± 3 bpm) compared to the baseline TTE test.

The average blood lactate levels during attrition time in the TTE test were similar during both baseline and post marching tests (6.64 ± 2.54 mmol · l^-1^ and 6.90 ± 3.6 mmol · l^-1^, respectively). Nevertheless, these lactate levels were far from what is expected during maximal aerobic performance (VO_2max_), which is above 8 mmol · l^-1^ [9]. Similarly, we did not find substantial differences in maximal HRs (HR_max_) at the time to exhaustion between the two phases (185 ± 6 bpm and 181 ± 8 bpm, respectively). During the baseline TTE test, however, these measures were achieved after a shorter period of time in comparison to the post-march test.

We suggest that the prior march increased the physiological strain during the following TTE test, as was expressed by higher HR values, and probably by higher body temperature and oxygen consumption (not measured) as part of the post exercise induced oxygen consumption (EPOC) phenomenon [10]. This accumulation of physiological strain prior the TTE test must have significantly decreased time to exhaustion. Nevertheless, since no HR_max_ according to age was measured during both baseline and post-march TTE tests, and no other markers of maximal aerobic or anaerobic performances were measured as was expressed by lactate levels (expected to be higher than 8 mmol · l^-1^), we suggest that subjective causes, as well as local muscle fatigue and the possible involvement of central nervous system (CNS) fatigue might have also been a contributor to exhaustion [11, 12].

It should be emphasized at this point, however, that although fatigue is a well-known phenomenon [13] and the phrase “exercised until exhaustion” is common, there is no unequivocal agreement on the fundamental nature of this process. Nevertheless, in the recent years this phenomenon has been further discussed, mostly due to the failure to fully understand the cause to exertional exhaustion using the common peripheral mechanisms. One of the significant examples is the presence of fatigue during exercise even though homeostasis is maintained [14]. It has been suggested that a “central governor” in the brain regulates our physical exertions. This governor integrates physiological information from throughout the body- core temperature, blood oxygenation, muscle signals and so on- along with other data based on previous experience and motivation and regulates how much strain can be achieved, probably in order to prevent reaching a dangerous state [14]. Future studies are required to continue exploring this phenomenon and its relation to various exercise types.

Analyzing the cognitive performances after the march using the VVT and the FCRT tests showed no differences in reaction time but a significantly higher number of incorrect identifications in the VVT test and similar trend in the FCRT test compared to baseline. The practical meaning of these results is that a 10 km military march under comfort conditions is sufficient to initiate a certain amount of haste that causes a decrease in accuracy. We assume that more extreme environmental conditions and/or longer march (to be studied) would contribute to worsening of these functions. Indeed, previous studies showed that changes in cognition as a result of prior physical load have been referred to intermediate factors, such as blood acidosis [15] central nervous system arousal [16], and changes in attention strategy [17]. It should be emphasized, regarding these results, that although some influences of exercise on cognitive performances were formerly described, no definite and practical conclusion emerged from these studies, and more importantly- no quantitative and practical measures were reported. This topic requires much more scientific attention, especially for operational and military training purposes.

This preliminary study has few limitations. The first limitation is the fact that it was conducted in laboratory conditions and not in field settings. We proposed that in order to create a more precise data base, without the environmental “noise” in field studies, we should first take measurements in laboratory conditions. We do find, however, future field studies, with various conditions as a supportive requirement. The second limitation is that the intensity of the march as was expressed by HR (average ranging between 110-127 bpm) was light-moderate for the young and fit subjects who participated in the study. Awareness of this fact is important when more intense pre-mission activities are going to be performed. Nevertheless, our purpose is to create extensive data that would include the influence of various pre-mission physical activities on selected performances. Further studies using different environmental and physical conditions and higher participation number will be required in order to create enough supported evidence-based data regarding the effect of pre-mission activities on performance. The third limitation is a possible influence of subjective motivation and different central exhaustion threshold levels. This should be considered in future similar studies aiming to predict performance after prior exercise loads. The fourth limitation is the fact that we have ignored the psychological stress that exists in every military related situation. These functions are not within the scope of this study and should definitely be considered and implemented in the future for both physical and cognitive prediction models.

## Conclusions

In conclusion, this study presents a practical approach to provide future assistance in predicting human performances, mostly military, in various conditions. The current results describe the influence of a moderate-intensity march under relatively comfort environmental conditions on selected military related physical and cognitive abilities. The most significant influence we found was on endurance aerobic exercise, where part of it seemed to be associated with mental fatigue and in haste during situations where quick decisions should be made. More studies using various conditions, both in the lab and in the field, are required in order to map the influence of various pre-mission physical activities and physical condition on combative performance. These data may contribute to preserving the field soldiers’ performance and health during both training and operational activities.

## Abbreviations

ATP-PCr: Adenosine triphosphate and phosphocreatine
CM: Centimeters
CNS: Central nervous system
EPOC: Post exercise induced oxygen consumption
FCRT: Four choice reaction time
HR: Heart rate
MIL: Milliliters
MSEC: Mili seconds
NS: Non significant
KG: Kilograms
KM: Kilometers
OBLA: Onset of blood lactate accumulation
RH: Relative humidity
SE: Standard error
SEC: Seconds
TTE: Time to exhaustion test
VO_2max_: Maximal oxygen uptake
VVT: Visual vigilance task.
